# Reducing harm from HIV/AIDS misconceptions among female sex workers in Tijuana and Ciudad Juarez, Mexico: A cross sectional analysis

**DOI:** 10.1186/1477-7517-9-35

**Published:** 2012-08-06

**Authors:** Angela M Robertson, Victoria D Ojeda, Lucie Nguyen, Remedios Lozada, Gustavo A Martínez, Steffanie A Strathdee, Thomas L Patterson

**Affiliations:** 1 Division of Global Public Health, Department of Medicine, University of California San Diego School of Medicine, Institute of the Americas, 10111 N. Torrey Pines Road, Mail Code 0507, La Jolla, CA, 92093-0507, USA; 2 Prevencasa, AC, Calle Baja California 7580, Zona Norte, 22000 , Tijuana, Mexico; 3 Salud y Desarrollo Comunitario de Ciudad Juárez, AC, Federación Mexicana de Asociaciones Privadas, Ciudad Juárez, Mexico; 4 Department of Psychiatry, University of California San Diego School of Medicine, 9500 Gilman Drive, Mail Code 0680, La Jolla, CA, USA

**Keywords:** HIV/AIDS, Knowledge, Female sex workers, Sex work harm reduction, Mexico

## Abstract

**Background:**

HIV prevalence is increasing among female sex workers (FSWs) in Mexico’s Northern border region, who experience multiple occupational risks. Improving vulnerable populations’ education, empowerment, and access to preventive services are important components of harm reduction strategies. Given the increasing interest in adapting harm reduction principles from drug use to sex work and other public health responses to the HIV epidemic, we used a sex work harm reduction framework to guide our investigation of FSWs’ HIV knowledge.

**Methods:**

From 2004–2006, FSWs aged ≥18 years in Tijuana and Ciudad Juarez participated in a behavioral intervention study and completed structured interviews. Measures included HIV knowledge assessment and factors within each domain of our theoretical framework for sex work harms: (1) socio-demographic factors that may lead to sex work, (2) sex work characteristics and behaviors that may lead to harm, and (3) mutually reinforcing harms that lead to sex work and result from it (e.g., drug abuse). Negative binomial regression identified factors independently associated with suboptimal HIV knowledge (i.e., incorrect responses during the HIV knowledge assessment).

**Results:**

Among 924 FSWs, the median proportion of incorrect responses was nearly one third (28% incorrect). Examination of item responses revealed misconceptions regarding specific transmission and prevention mechanisms, including prevention of mother to child transmission. Suboptimal HIV knowledge was independently associated with older age, lower education, living in Tijuana (vs. Ciudad Juarez), inconsistent condom use for vaginal sex with male clients, and lacking prior HIV testing.

**Conclusions:**

Our application of a sex work harm reduction framework to the study of FSWs’ HIV knowledge is an important first step in enhancing HIV prevention efforts in Northern Mexican border cities. Our findings imply that interventions should identify and discredit local HIV misconceptions to improve knowledge of specific HIV transmission routes and self-protective strategies (e.g., condom negotiation). Interventions will require materials appropriate for women from diverse socio-economic backgrounds and may benefit from innovative harm reduction approaches such as peer education and outreach.

## Introduction

Female sex workers (FSWs) face many physical and mental health harms through their occupation, including risk of disease acquisition (e.g., HIV and other sexually transmitted infections; STIs), physical and sexual violence, discrimination, criminalization and exploitation [1]. Increasingly, researchers and practitioners who work with FSWs are calling for a broader application of the harm reduction agenda from drug use to sex work and other public health responses to the HIV epidemic [1,2]. Using a harm reduction perspective, Cusick recently conceptualized three types of sex work harms: (1) vulnerabilities and structural factors that may lead to sex work, such as poverty, low education, lack of employment opportunities and other socio-demographics; (2) harms introduced by sex work, including HIV/STI transmission, physical and sexual abuse, and other harms to health and well-being that result from sex work activities; and (3) mutually reinforcing harms such as drug addiction that may simultaneously contribute to involvement in sex work (e.g., to earn money for drugs) and result from engagement in it (e.g., drug abuse to cope with the physical and emotional stress of the occupation) [2]. Potential components of harm reduction interventions for FSWs could include increasing vulnerable FSWs’ education, empowerment, access to prevention and healthcare services, occupational health and safety policies, decriminalization of sex work and other human rights approaches [1]. As a starting point, educational components of harm reduction programs can work with local populations of FSWs to increase knowledge of HIV/STIs and protective behaviors, dispel misconceptions, decrease risk behaviors, and improve access to services [3].

Knowledge and comprehension of health risk and protective behaviors, as well as access to information, are key components of many health behavior models [4-6]. Having an accurate understanding of HIV/STI transmission is associated with increased condom use [7,8], and misinformation regarding transmission risk from casual contact (e.g., kissing, sharing a drinking glass) has been associated with stigmatizing attitudes [9]. Therefore, increasing knowledge of HIV transmission routes and risk and protective behaviors plays an important role in many HIV-prevention interventions [10-12]. However, individuals with high knowledge often fail to protect themselves and others from HIV/STIs due to factors occurring at the interpersonal, community, and social/structural levels [6] as emphasized by socio-ecological models [13] and harm reduction perspectives [2]. Designing effective interventions to increase HIV knowledge and reduce harms associated with sex work require an assessment of populations’ baseline knowledge and their access to accurate, timely health information [14]. Once HIV knowledge has been assessed in a population, harm reduction strategies such as peer education and community outreach can be developed [1,3].

While Mexico has a low overall HIV prevalence [15], social and structural vulnerabilities and high risk sexual- and drug-related risk behaviors have resulted in a dynamic HIV epidemic along Mexico’s border with the United States [16-19]. Of particular concern is the elevated prevalence of HIV among FSWs and injection drug users (IDUs) in Tijuana, Baja California and Ciudad Juarez, Chihuahua, which border San Diego, California and El Paso, Texas, respectively. As Mexico’s two largest border cities, both Tijuana and Ciudad Juarez are situated on major drug-trafficking routes supporting large populations of drug users [20]. Both cities have *zonas rojas* (red light districts) supported by sex tourism from the United States and elsewhere in Mexico [21]. Recent estimates place the numbers of FSWs at 9,000 in Tijuana [16] and 4,100 in Ciudad Juarez [22]. HIV prevalence among FSWs in both cities was recently estimated at 6% [19].

In Mexico, knowledge of HIV transmission remains low in some communities and segments of the health care sector [23-28]. Correct knowledge may co-exist with incorrect beliefs [29,30], as research has shown in other countries in Latin America [31,32]. Although students in some Mexican public schools are currently exposed to accurate messages about HIV transmission and prevention, adolescents may simultaneously hold misconceptions regarding protective behaviors [27]. In one sample of urban youth, although most could identify transmission routes, many held misconceptions regarding correct condom use, the distinction between HIV and AIDS, and the possibility of acquiring HIV through casual contact with patients in healthcare settings [29]. Furthermore, the exact relationship between HIV knowledge and engagement in risk behavior may vary by socio-demographic factors, including gender [27], requiring further study for interventions with high risk populations. A qualitative study in Tijuana suggested that FSWs have low knowledge regarding the proper use of condoms to prevent HIV [33], yet empirical data are lacking on FSWs’ overall awareness of HIV transmission routes and prevention strategies. Data on HIV knowledge and other factors that may predispose FSWs in the U.S.-Mexico border region are lacking. Given the potential benefit of understanding baseline HIV knowledge as a starting point in the development of a comprehensive sex work harm reduction agenda, we sought to identify specific HIV misconceptions and correlates of suboptimal HIV knowledge among FSWs in Tijuana and Ciudad Juarez, Mexico. We based our theoretical framework on socio-ecological models [13] and Cusick’s conceptualization of sex work harms [2]. We hypothesized that suboptimal HIV knowledge would be associated with vulnerabilities or socio-demographic factors leading to sex work (e.g., low education), harmful behaviors or factors resulting from sex work (e.g., inconsistent condom use), and mutually reinforcing harms (e.g., recent drug abuse). Our findings may contribute to the development of an enhanced sex work harm reduction framework for the U.S.-Mexico border region including recommendations for the development of an educational intervention to increase FSWs’ knowledge and empowerment with respect to HIV prevention strategies.

## Methods

### Study population

Outreach workers and health clinic staff recruited FSWs in Tijuana (*N* = 474) and Ciudad Juarez (*N* = 450) into a behavioral intervention study that was designed to increase condom use, as previously described [21,34]. Eligibility criteria included being ≥18 years of age, self-identifying as a FSW (i.e., having traded sex for drugs, money, or other material goods in the past month), having had unprotected vaginal or anal sex with ≥1 client in the past month, reporting HIV-negative status, and providing informed consent. Women were excluded if they reported always using condoms with all of their male clients, which was a main outcome of interest for the intervention study.

### Data collection

Baseline data were collected from 2004–2006 by trained, Spanish-speaking female counselors. Interviews lasted approximately 45 minutes and were conducted in private clinic rooms and outreach offices in *zona roja* neighborhoods. Study instruments were based on extensive pilot-testing in Tijuana [35], and the baseline questionnaire was translated into Spanish and back-translated into English [36,37]. Participants were compensated U.S. $30 for their time. Institutional review boards in Mexico and the United States approved all study protocols.

### Measures

Our dependent variable, HIV unawareness, henceforth referred to as “suboptimal HIV knowledge,” was assessed using an 18-item questionnaire developed to be consistent with a longer, previously validated HIV knowledge scale [14]. The 18-item scale contains true/false statements regarding routes of HIV transmission and self-protection strategies, as listed in Table 1. Other measures were organized according to the three broad domains of sex work harms conceptualized in our theoretical framework: (1) *socio-demographic factors that may lead to sex work* included age, educational attainment (years of school completed), born in study site (vs. migrant), current residence in Tijuana (vs. Ciudad Juarez), duration of residence in study site (years), and ability to speak some English; (2) *sex work characteristics and health behaviors that could lead to harm* included experience in sex work (years involved), municipal registration as a sex worker (Tijuana only), nationality of clients (Mexican only vs. some U.S. clients), type of FSW (e.g., street worker, dance hostess, bar maid, or brothel worker), recent condom use for vaginal sex with male clients (past 6 months), recent STI diagnosis (past 6 months), history of HIV testing (lifetime), HIV positivity, and recent HIV counseling (past 6 months); and (3) *drug abuse factors as a type of mutually reinforcing harm* included recent injection drug use (past 6 months), using drugs before sex (past month), and having IDU sex partners (past month). 

**Table 1:** **Incorrect responses to HIV knowledge questionnaire**^**a**^**among 924 FSWs in Tijuana and Ciudad Juarez**

**Items**^**b**^	***N***	**Incorrect (%)**
1. Coughing and sneezing DO NOT spread HIV. (T)	922	526 (57.0 %)
2. A person can get HIV by sharing a glass of water with someone who has HIV. (F)	922	172 (18.7 %)
3. Pulling out the penis before a man climaxes keeps a women from getting HIV during sex. (F)	921	290 (31.5 %)
4. A woman can get HIV if she has anal sex with a man. (T)	923	75 (8.1 %)
5. Showering or washing one’s genitals after sex keeps a person from getting HIV. (F)	923	195 (21.1 %)
6. All pregnant women infected with HIV will have babies born with AIDS. (F)	922	800 (86.8 %)
7. People who have been infected with HIV quickly show serious signs of being infected. (F)	923	293 (31.7 %)
8. There is a vaccine that can stop adults from getting HIV. (F)	913	243 (26.6 %)
9. People are likely to get HIV by deep kissing, putting their tongue in their partner’s mouth, if their partner has HIV. (F)	923	345 (37.4 %)
10. A woman cannot get HIV if she has sex during her period. (F)	923	294 (31.9 %)
11.There is a female condom that can help decrease a woman’s chance of getting HIV. (T)	918	361 (39.3 %)
12. A natural skin condom works better against HIV than does a latex condom. (F)	920	237 (25.8 %)
13. A person will NOT get HIV if she or he is taking antibiotics. (F)	922	173 (18.8 %)
14. Having sex with more than one partner can increase a person’s chance of being infected with HIV. (T)	924	46 (5.0 %)
15. Taking a test for HIV one week after having sex will tell a person if she or he has HIV. (F)	922	375 (40.7 %)
16. A person can get HIV by sitting in a hot tub or a swimming pool with a person who has HIV. (F)	922	221 (24.0 %)
17. A person can get HIV if having oral sex, mouth on penis, with a man. (T)	921	186 (20.2 %)
18. Using Vaseline or baby oil with condoms lowers the chance of getting HIV. (F)	921	140 (15.2 %)
Median Incorrect Proportion (IQR) on HIV Knowledge Scale	0.28	(0.17–0.39)

^a^*Carey and Schroder’s HIV Knowledge Questionnaire (2002)*.

^*b*^*Correct answers appear in parentheses after each item (T = true; F = false)*.

### Statistical analysis

To assess suboptimal HIV knowledge, our outcome of interest, we first assessed FSWs’ responses to individual items in the HIV knowledge questionnaire. We obtained the proportion of incorrect responses on the overall set of HIV knowledge questions by summing incorrect responses for each participant and dividing by the total number of questions answered by each participant. To identify factors associated with suboptimal HIV knowledge, we modeled the proportion of incorrect responses using negative binomial regression with the total number of incorrect responses as the outcome variable and the log of the total number of questions answered as the offset term. Univariate and multivariate negative binomial regression models identified factors associated with suboptimal HIV knowledge within the three domains of our theoretical framework described above. To identify variables independently associated with suboptimal HIV knowledge, multivariate models were developed using a manual procedure in which all variables of interest attaining significance levels <10% in univariate models were considered. The final model retains only variables statistically significantly associated with suboptimal HIV knowledge, the dependent variable. We did not find evidence of interactions or multicollinearity in the final model.

## Results

### HIV knowledge among FSWs

Among 924 FSWs, overall HIV knowledge was moderate, with women answering incorrectly to a median of 28% of questions (median incorrect rate: 0.28; interquartile range [IQR]: 0.17–0.39). As shown in Table 1, responses to individual items on the HIV knowledge scale varied: 87% of women incorrectly answered that HIV-positive mothers will give birth to infants with AIDS, and 57% of women incorrectly answered that coughing and sneezing transmit HIV. Although most women (>90%) were aware of two important modes of sexual HIV transmission (e.g., anal sex and multiple sexual partners), approximately one-third of women incorrectly answered items with important implications for HIV prevention, including the following misconceptions: kissing can transmit HIV (37% incorrect), having sex during a woman’s menstrual period can prevent HIV transmission (32% incorrect), and withdrawal of the penis preceding male climax can prevent transmission (32% incorrect). Furthermore, 32% of women did not know that HIV symptoms do not always quickly manifest in infected persons, 39% were not aware of the existence of female condoms that are efficacious for preventing HIV transmission, and 41% incorrectly responded that HIV testing can identify the presence of infection immediately following unsafe sex.

### Characteristics of FSWs associated with suboptimal HIV knowledge

Characteristics of our sample of FSWs, who had a median age of 32 years (IQR: 26–39) and educational attainment of 6 years (IQR: 4–8), are shown in Table 2. We identified factors associated with suboptimal HIV knowledge within each of the three domains of our theoretical framework. First, within the socio-demographic domain, we found that suboptimal HIV knowledge (i.e., increasing rate of incorrect responses on the HIV knowledge questionnaire) was associated with increasing age (Unadjusted Risk Ratio [*RR*] = 1.10 per 10 years; 95% confidence interval [CI]: 1.06–1.13) and residing in Tijuana as compared to Ciudad Juarez (*RR* = 1.17; 95% CI: 1.10–1.25). Increasing education (*RR* = 0.95 per year increase in educational attainment; 95% CI: 0.94–0.96), speaking English (*RR* = 0.87; 95% CI: 0.81–0.95), and having clients from the United States (*RR* = 0.91; 95% CI: 0.85–0.97) were protective against suboptimal HIV knowledge (i.e., they were associated with higher HIV knowledge in univariate models). Second, within the sex work characteristics and behaviors domain, we found that having any positive STI diagnosis in the past six months was associated with suboptimal HIV knowledge (*RR* = 1.09; 95% CI: 1.02–1.16). We also found that “always” or “often” using condoms for vaginal sex with male clients (*RR* = 0.85; 95% CI: 0.79–0.91), having had any prior HIV testing (*RR* = 0.92; 95% CI: 0.86–0.98), and having HIV counseling in the past six months (*RR* = 0.82; 95% CI: 0.73–0.92) were protective against suboptimal HIV knowledge. Regarding drug abuse, the third domain of our theoretical framework, we found that recent drug use before or during sex was marginally associated with suboptimal HIV knowledge (*RR* = 0.93; 95% CI: 0.87–1.00).

**Table 2:** **Factors associated with suboptimal HIV knowledge**^**a**^**among 924 FSWs in Tijuana and Ciudad Juarez, Mexico**

**Baseline characteristics**	***N*(%)**^**b**^**or Median (IQR)**^**c**^	**Unadjusted risk ratio estimate**	**95 % Confidence interval**
**Socio-demographics**			
Age (per 10 years)**	3.2 (2.6–3.9)	1.10	1.06–1.13
Education (in school years completed)**	6 (4–8)	0.95	0.94–0.96
Born in study location (vs. migrant)*	361 (39 %)	0.94	0.88–1.01
Currently resides in Tijuana (vs. Ciudad Juarez)**	474 (51 %)	1.17	1.10–1.25
Duration residing in study location (in years)	13 (6–26)	1.00	1.00–1.00
Speaks some English**	237 (26 %)	0.87	0.81–0.95
**Sex Work Characteristics & Behaviors**			
Experience in sex work (in years)	4 (2–10)	1.00	1.00–1.01
Registered with Tijuana Municipal Health Services (Tijuana only) *	181 (44 %)	0.91	0.83–1.01
All clients are Mexicans**	267 (29 %)	1.09	1.02–1.17
Has clients from the US**	634 (69 %)	0.91	0.85–0.97
Street worker	512 (55 %)	0.96	0.90–1.03
Dance hostess	196 (21 %)	1.06	0.98–1.15
Bar maid	144 (16 %)	1.03	0.95–1.12
Brothel worker	24 (3 %)	0.88	0.69–1.11
Often/always uses condoms for vaginal sex with male clients (past month)**	399 (43 %)	0.85	0.79–0.91
Any positive STI diagnosis (past six months)**	340 (37 %)	1.09	1.02–1.16
Has had prior HIV testing**	471 (51 %)	0.92	0.86–0.98
HIV positive	55 (6 %)	1.11	0.97–1.26
Counseling past six months**	73 (8 %)	0.82	0.73–0.92
**Drug Use Behaviors**			
Ever injected drugs (past six months)	124 (13 %)	0.94	0.85–1.04
Used illegal drugs before/during sex (past month)*	297 (32 %)	0.93	0.87–1.00
Had an IDU partner (past month)	194 (21 %)	0.97	0.90–1.06

^*a*^*Incorrect responses on Carey and Schroder’s HIV Knowledge Questionnaire (2002).*

^*b*^*Certain percentages may reflect denominators smaller than N = 924. Except as specifically noted, these discrepancies are due to missing data.*

^*c*^*Inter-Quartile Range (IQR).*

*p ≤0.10; **p <0.05.

### Factors independently associated with suboptimal HIV knowledge among FSWs

As shown in Table 3, three factors from the socio-demographic domain of our theoretical framework were independently associated with suboptimal HIV knowledge in our final multivariate regression model: older age (Adjusted Risk Ratio [*ARR*] = 1.06 per 10 years; 95% CI: 1.02–1.09), living in Tijuana vs. Ciudad Juarez (*ARR* = 1.25; 95% CI: 1.17–1.33), and educational attainment (*ARR* = 0.96 per year increase in educational attainment; 95% CI: 0.95–0.97). Two factors within the sex work domain were associated with suboptimal HIV knowledge: often or always using condoms for vaginal sex with male clients (*ARR* = 0.91; 95% CI: 0.85–0.97) and having any prior HIV testing (*ARR* = 0.91; 95% CI: 0.85–0.97). No factors from the drug abuse domain of theoretical framework were independently associated with suboptimal HIV knowledge.

**Table 3:** **Factors independently associated with suboptimal HIV knowledge**^**a**^**among 913 FSWs in Tijuana and Ciudad Juarez, Mexico**

**Variable**^**b**^		**Adjusted risk ratio estimate**	**95 % Confidence interval**
**Socio-demographics**			
	Age (per 10 years)	1.06	1.02 – 1.09
	Currently resides in Tijuana (vs. Ciudad Juarez)	1.25	1.17 – 1.33
	Education (in school years completed)	0.96	0.95 – 0.97
**Sex Work Characteristics & Behaviors**			
	Has had prior HIV testing	0.91	0.85 – 0.97
	Often/always uses condoms for vaginal sex with male clients (past month)	0.91	0.85 – 0.97

^***a***^*Incorrect responses on Carey and Schroder’s HIV Knowledge Questionnaire (2002).*

^*b*^*All variables checked for multicollinearity and interaction; p < .05 for all values.*

## Discussion

We found that suboptimal HIV knowledge was associated with factors representing two of the three domains of Cusick’s framework for understanding sex work harms. As we hypothesized, socio-demographic factors that may lead to sex work, and sex work characteristics and behaviors that could lead to harm [2] were associated with suboptimal HIV knowledge. Within the first domain (i.e., socio-demographics), we found that suboptimal HIV knowledge was associated with older age, lower education, and living in Tijuana (vs. Ciudad Juarez). Although educational attainment has increased in Mexico in recent years [38], HIV education had not been systematically implemented in public schools across the country at the time when our participants would have been in school [39]. The median age (32 years) and educational attainment (6 years) of FSWs in our study suggests that older women may have missed any early HIV/AIDS messages in schools and received information from community sources (e.g., media coverage, health department campaigns, etc.) as well as misinformation fueled by social norms. Low education in our sample could also be a marker for poor health literacy [10], and other unmeasured socio-economic and structural factors. Our finding that women in Tijuana had greater suboptimal HIV knowledge could reflect important differences in educational programs, health department campaigns, and outreach to FSWs between the two cities. Taken together, these findings suggest that harm reduction interventions for FSWs in the U.S.-Mexico border region should make special efforts to reach older, less educated women in Tijuana with information available through linguistically and culturally appropriate, non-reading-based educational media (e.g., radio, television, and peer contact) [40-43]. In particular, peer education and outreach, in which women from diverse socioeconomic and cultural backgrounds could be trained to educate and provide information to their peers, could also help to address this programmatic need [3].

Within the second domain of Cusick’s conceptualization of sex work harms, sex work factors and behaviors that directly lead to harm, we found that suboptimal HIV knowledge was associated with limited healthcare access (e.g., lacking prior HIV testing) and failure to consistently use self-protective health behaviors (e.g., not always using condoms for vaginal sex with male clients). These findings help expand a small body of literature on HIV knowledge, health care access, and health behaviors among FSWs internationally. Accurate reproductive health knowledge was associated with having health insurance among Thai FSWs [44], and greater contact with health care professionals among Italian FSWs [45]. In Turkmenistan, street-based FSWs had the lowest HIV knowledge and rarely initiated condom use with clients [46]. It is possible that women lacking access to HIV testing and other healthcare services may not receive as much HIV prevention information as women not accessing services. The World Health Organization’s HIV/AIDS Sex Work Toolkit specifically calls for education to increase the knowledge and use of voluntary counseling and testing (VCT) for HIV and other healthcare services [3]. Our finding that suboptimal HIV knowledge was associated with not always using condoms for vaginal sex with clients could also reflect poor access to HIV prevention services, including those that distribute condoms and prevention information. This finding is also supported by many health behavior theories [4-6] and global recommendations [11,12].

Within the third domain of our framework, we did not find support that any mutually reinforcing harms of sex work (e.g., drug abuse) are related to HIV knowledge. Although recent drug use before or during sex was associated with HIV knowledge in our univariate analysis, this association did not persist in the final model controlling for factors in the other two domains of Cusick’s framework [2]. The socio-demographic and sex work factors/behaviors that we identified may have stronger relationships with HIV knowledge than mutually reinforcing harms. Additional research may be needed to investigate and elaborate upon this third domain of sex work harms as well as other higher-level, social and structural factors impacting HIV knowledge, as highlighted in socio-ecological models [13]. Nevertheless, our study of HIV knowledge yielded several findings with important implications for HIV prevention and the broader sex work harm reduction agenda in cities along Mexico’s Northern border with the United States.

Understanding the extent of HIV knowledge and misconceptions is essential in the development of sex work harm reduction interventions for this population [1,2]. By examining FSWs’ responses to individual HIV knowledge scale items, we found that accurate knowledge of HIV transmission and prevention often coexists with misconceptions. For example, although >90% of women correctly identified multiple sex partners and anal sex as risk behaviors for HIV transmission, 87% incorrectly believed that HIV is *always* transmitted from pregnant mothers to their offspring, a misconception that may reflect a lack of awareness of prevention of mother-to-child-transmission services. Women’s knowledge of the role of latex condoms in HIV transmission was quite high: 85% answered that oil-based lubricants, which can degrade latex and possibly cause condoms to tear or break during sex [47], do *not* help lower one’s chance of acquiring HIV. However, 26% incorrectly believed natural skin condoms, which are ineffective in preventing transmission of HIV and other STIs [47], to be superior to latex condoms. Nearly one third of our sample incorrectly believed that HIV-positive persons quickly show symptoms, which is similar to findings from a U.S. study in which one third of Mexican-born adults did not believe that infected individuals can feel and look healthy [48].

The co-existence of correct and incorrect knowledge has been documented in other settings and may reflect preexisting misunderstandings of disease transmission combined with more recent exposure to scientific/medical information [29,31,32,48,49]. HIV misconceptions have been shown to displace correct HIV knowledge, giving some individuals a false sense of security while engaging in risk behaviors, increasing individuals’ anxiety regarding susceptibility and testing, promoting discrimination and stigma against HIV-positive persons, and impacting social interactions and activities [50,51]. In order to best target harm reduction and health education services to FSWs, additional research may be needed on the profiles of women holding specific types of misconceptions. In addition to providing accurate information on HIV transmission and prevention that is appropriately targeted to specific FSW populations, sex work harm reduction interventions should address misconceptions by carefully working with FSWs to identify and discredit local sources of misinformation including social norms that support stigmatizing behaviors or inappropriate prevention strategies [52]. Peer education and outreach approaches have successfully increased HIV/STI knowledge among FSWs and their male clients internationally [53-56], prompting the World Health Organization to include peer education strategies in the HIV/AIDS Sex Work Toolkit [3].

Our study was limited by several factors. First, we relied on cross-sectional data, preventing elucidation of temporal changes in HIV knowledge or causal relationships. Additional research is needed to help discern the temporal and causal relationships between FSWs’ suboptimal HIV knowledge, access to accurate HIV information, access to healthcare including VCT services, and condom use with clients. Second, since our sample included only FSWs recruited through outreach and clinical staff, our findings cannot be generalized to the adult population in these cities. Third, the HIV knowledge instrument we used was not designed for FSWs’ unique socio-cultural or linguistic contexts. Although the HIV knowledge questionnaire has been successfully modified for relevance for other populations (e.g., by adding colloquial terms) [57], efforts are needed to assess the measurement of HIV knowledge in these bilingual, culturally diverse U.S.-Mexico border communities [36,37,58,59]. Fourth, our data were collected from 2004–2006. While it is possible that HIV knowledge may have improved recently due to increased public health attention and resources directed toward high risk populations in this region, some misconceptions may persist. Any intervention with FSWs should begin with an accurate, up-to-date needs assessment including measures of HIV knowledge [60].

Finally, we must acknowledge that awareness is only one component of health behavior change. For FSWs in resource-constrained settings, the immediacy of economic and survival concerns, particularly in light of the recent drug violence in Mexico and declining tourism in Mexico [61,62], may override the importance of HIV knowledge or the ability to act upon it. Additional structural-level factors that increase FSWs’ vulnerability to HIV and inability to act on HIV prevention knowledge include abuse by police [63], poverty, lack of condom access [64], and poor access to healthcare services including treatment for ulcerative STIs that are associated with increased risk of HIV [17]. These structural factors, which may require further research and conceptualization using socio-ecological models [13], will likely impact the ultimate efficacy of harm reduction interventions for FSWs [65].

## Conclusions

Knowledge is one essential component of reducing the harms from risky health behaviors and occupations such as sex work. As a first attempt to understand HIV knowledge among FSWs in Tijuana and Ciudad Juarez, our study used a sex work harm reduction framework to identify factors associated with suboptimal HIV knowledge. Our findings have important implications for the development of HIV prevention and sex work harm reduction interventions in the U.S.-Mexico border region. Qualitative research methods can help programs identify local HIV misconceptions and norms supporting such beliefs, thereby enabling programs to be tailored to unique community needs with educational materials that are linguistically and culturally appropriate and accessible for FSWs and their male clients in border cities [21,33,35]. Interventions can leverage existing HIV information campaigns in Mexico [23,27] and adopt innovative harm reduction approaches such as peer education and outreach to reach older, less educated women living in Tijuana. Programs should also ensure that women are aware of and able to access condoms and VCT services. Increasing HIV knowledge through programs that consider the context of FSWs’ lives will support ongoing prevention efforts to stem the HIV epidemic among high risk populations in the US-Mexico border region.

## Abbreviations

FSW: Female sex worker; HIV: Human immunodeficiency virus; STI: Sexually transmitted infections; IDU: Injection drug user; IQR: Interquartile range; RR: Unadjusted risk ratio; CI: 95% confidence interval; ARR: Adjusted risk ratio; VCT: Voluntary counseling and testing.

## Competing interests

The authors declare that they have no competing interests.

## Authors’ contributions

AR contributed to manuscript development, and editing. VO, TP and SS contributed to study design, analyses, and editing. LN ran analyses, wrote the methods and results section, and contributed to editing. RL and GM contributed to study design, data collection and manuscript editing, and served as a liaison to our Mexican counterparts. TP was PI and contributed heavily to direction of analyses, manuscript drafting, and editing. All authors read and approved the final manuscript.
